# *Hif3α* Plays Key Roles in the Progression of Alzheimer’s Disease Caused by Circadian Rhythm Disruption through Regulating the m^6^A/KDM3A/TGF-β1 Axis

**DOI:** 10.3390/biology13060412

**Published:** 2024-06-04

**Authors:** Xinrui Li, Zhengkun Han, Huiying Li

**Affiliations:** 1 Beijing National Day School, Beijing 100062, China; xinruili8888@163.com; 2 Beijing Key Laboratory of Food Processing and Safety in Forest, College of Biological Sciences and Technology, Beijing Forestry University, Beijing 100083, China; zhengkunh1005@126.com

**Keywords:** circadian rhythm disruption, Alzheimer’s disease, m^6^A methylation, *Hif3α* gene

## Abstract

**Simple Summary:**

Circadian rhythms manipulate various physiological functions to help organisms adapt to environmental variations in a 24 h cycle. However, there are very few studies identifying key sensitive genes linking disrupted circadian rhythms to the progression of Alzheimer’s disease (AD), especially elucidating the important role of the key genes at the epigenetic molecular level. Thus, the present study constructed a disrupted circadian rhythm model for accelerated progression of AD, and the results showed increased cellular oxidative stresses, reduced antioxidant capacities, and damage to neuronal cells and brain function in model mice. Then, an increase in RNA m6A levels in mice’s brains due to disturbed circadian rhythms was observed, and the special m6A methylation site of the Hif3α gene is 3632 was proven to be involved. This leads us to propose a potential mechanism for the observed malignant progression of AD. Last but not the least, protein expression of KDM3A and TGF -β1 decreased as HIF3A expression increased, indicating that the three co-existing factors might affect disease progression caused by disrupted circadian rhythms.

**Abstract:**

Disrupted circadian rhythms are associated with the onset of chronic diseases and impairments, including cancer, diabetes, and hypertension. However, whether circadian disruptions accelerate the progression of Alzheimer’s disease and the respective pathway remains unclear. In this study, we constructed animal models using male C57BL/6N and APP/PS1 mice. Irregular illumination during sleeping hours was administered to the mice in our intervention groups to consistently disrupt their circadian rhythms. The impact of the intervention was evaluated through body weight tracking, cerebral index determination, histopathological staining, and biochemical marker analysis. Transcriptomic sequencing identified critical genes, with the data subsequently validated using RNA m^6^A detection and site analysis. The evaluations revealed that circadian disruptions impaired normal weight gain, liver and kidney functions, neuronal cells, and overall brain function. Transcriptomic sequencing data revealed a trend of elevating expression of *Hif3α* mRNA in the intervention groups. Further analysis of specific gene sites revealed that m^6^A methylation of the *Hif3α* gene at m^6^A site 3632 primarily drove the observed variations in HIF3A protein expression in our model. Furthermore, the expression of proteins in PC12 cells, N2a cells, and mice brains validated that an increase in HIF3A expression decreased KDM3A and TGF-β1 protein expression. Our study reveals a hitherto unknown pathway through which the disruption of circadian rhythms, by triggering m^6^A methylation at m^6^A site 3632 in the *Hif3α* gene, leads to the initiation and acceleration of AD. These findings provide valuable insights and guidelines for treating AD patients and enhancing caregiving by professionals.

## 1. Introduction

The circadian rhythms, also known as biological clocks, direct the within-cycle operations of organisms over an approximately 24 h light/dark period. These internal timekeepers manage a variety of cellular processes, physiological functions, and behavior, enabling organisms to respond promptly to cyclic environmental fluctuations inherent in the passing of day and night [1]. However, while circadian rhythms are capable of guiding us to recalibrate over time, extended periods of misalignment with environmental cues potentially trigger the emergence of chronic diseases. These include type II diabetes, hyperlipidemia, hypertension, stroke, and various forms of cancer. Research using a rat model has demonstrated that exposure to light during the nighttime can disrupt the pancreatic clock, compromising the survival and function of β-cells and increasing the susceptibility to type II diabetes [2]. Turek unveiled an important link between *Clock* gene mutations and deviations in eating timing and glucose and lipid metabolism, underlining the potential for these mutations to lead to metabolic disorders associated with obesity and diabetes [3]; this supports the notion that preserving normal circadian rhythms is essential to maintaining health on both the molecular and behavioral fronts. As for cardiovascular diseases, alterations to the circadian clock in cardiac tissues have been observed in individuals diagnosed with these conditions, and abnormal circadian rhythms have been confirmed to amplify cardiovascular risk factors [4]. Furthermore, dysregulation of daily behavioral cycles may stimulate tumor growth: some circadian genes may even function as oncogenes or tumor suppressor genes, playing crucial regulatory roles in tumor development [5]. Thus, circadian genes are crucial in preserving human health. Disruptions to circadian rhythms or malfunctioning of circadian genes could instigate or exacerbate the progression of chronic diseases.

Alzheimer’s disease (AD), a chronic neurodegenerative disorder, is a leading cause of dementia in the elderly population. Patients diagnosed with AD undergo a progressive decline over the disease course in memory and other cognitive abilities, including learning and identification capabilities [6]. Hallmarks of AD consist of amyloid-β peptide (Aβ protein) aggregating into plaques within the brain tissue, along with neurofibrillary tangles resulting from alterations to the neuronal cytoskeleton due to hyperphosphorylation of Tau protein within the microtubules [6]. The primary regions afflicted by the disease comprise the medial temporal lobes and associated cortical structures [6]. Following initial diagnosis, the average life expectancy of a patient with AD ranges from 8 to 12 years. The mild stage is typically sustained for approximately 3 years, whereas the moderate and severe stages can persist for a period of 2–10 years and 8–10 years, respectively. This lengthy disease trajectory imposes tremendous socio-economic strain on the patients and their families [6]. There is no known cure for AD to date. Patients suffering from AD worldwide rely on two types of approved medications: cholinesterase inhibitors and N-methyl-D-aspartate (NMDA) antagonists. Unfortunately, while effective in symptom management, these drugs are unable to halt disease progression or provide a curative solution [7]. Given the weighty burden that AD places on healthcare systems, families, and government budgets, and the mounting distress of patients, we face a significant future challenge. The continuous care required by AD sufferers, particularly in moderate and severe stages, necessitates efforts to improve the caregivers’ diminished quality of life, alongside protecting the patients.

The genesis and functionality of proteins in humans are fundamentally linked to genetic materials—DNA and RNA—which are intricately guided by a variety of epigenetic modifications [8]. Among the numerous perceived modifications, methylation processes and their consequent effects have drawn considerable interest, and RNA methylation, when compared to DNA methylation, exhibits a significant advantage—it manipulates gene attributes at the post-transcriptional stage, which amplifies its applicability [8]. Consequently, RNA methylation has become a major focal point in recent research discussions. N6-Methyladenosine (m^6^A) is the most widespread methylated base in the mRNA and long non-coding RNA (lncRNA) of eukaryotes [8]. Studies have revealed that RNA m^6^A performs crucial regulatory functions across key biological processes and a range of diseases. In addition to influencing metabolism and the proliferation of tumors, it has been identified in the modification and control of mRNA output, playing a significant role in the regulation of circadian rhythms [9,10]. This regulatory function of RNA m^6^A is revealed in its interactions with various cellular metabolic signals, but details of these molecular mechanisms are yet to be fully understood. There is compelling evidence suggesting that RNA m^6^A increases in frequency in brain cell genes as we age [11] and that it plays critical roles in processes such as neuron development and memory formation [12]. Moreover, abnormal regulation of RNA m^6^A might lead to age-associated neurodegenerative diseases. While more research concentrates on Parkinson’s disease, some studies also propose a possible association between aberrant m^6^A levels and AD [13].

In the context of upholding circadian rhythms and enforcing standardized treatments, the proactive management of AD progression and the execution of effective, standardized treatments emerge as vital factors for a better application of societal resources and to protect the physical and emotional well-being of both caregivers and patients. Emerging evidence has established a correlation between the progression of AD, aging, and disruptions in circadian rhythms [14,15]. Yet, studies examining the impact of circadian rhythms on AD progression are markedly limited, and investigations into the molecular mechanisms governing this disease are even rarer. This scarcity calls for increased scientific exploration and validation from researchers. Accordingly, in order to evaluate the negative effects of circadian rhythm disruptions on AD progression, as well as to elucidate the particular mechanism in this course, we conducted the present research, which analyzed the impact of circadian disruptions on healthy mice and those suffering from AD. We compared gene expression levels in brain tissues between groups, singled out candidate genes, and further analyzed key RNA m^6^A methylation sites associated with those genes. Lysine demethylase 3A (KDM3A) operated as a demethylase, modulating transcription by removing repressive demethylation marks from the histone H3’s lysine 9 [16]. Meanwhile, transforming growth factor-β (TGF-β1), a cell transformation factor, regulated cellular immune function, fibrosis, and cancer progression [17]. Remarkably, both KDM3A and TGF-β1 had protective roles under hypoxic organ conditions and contributed to responses to neuroinflammation [18,19,20,21]. Our research findings lead us to propose that circadian rhythm disruption might exacerbate AD development via the methylation of key gene m^6^A, which might be related to KDM3A and TGF-β1. By delving into the molecular mechanisms through which the disruption of circadian rhythms may contribute to the malignant progression of AD, we offer insights that could enhance AD treatment and professional caregiving.

## 2. Materials and Methods

### 2.1. Animal Model Construction

Male C57BL/6N mice and APP/PS1 transgenic AD mice (n = 5 in each group) aged 8–10 weeks were purchased from Nanjing Jicui Biological Company and maintained at SPF-grade standards. The mice were kept at (25 ± 0.5) °C with 55% humidity in a cycle of darkness from 8:00 a.m. to 8:00 p.m. and light from 8:00 p.m. to 8:00 a.m. The intervention started after 7 days of acclimatization. This study was approved by the Zhongyan Zichuang Animal Ethics Committee of Beijing Changping District Life Science Park (Experimental Ethics Approval No. ZYZC202307008S).

**Figure 1:**
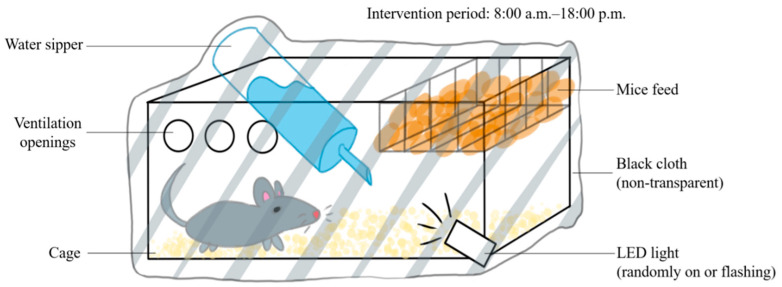
Schematic diagram of the circadian rhythm disruption treatment method.

The C57BL/6N and APP/PS1 mice were randomly assigned into 4 groups: normal–control, normal–circadian disruption, AD–control, and AD–circadian disruption, each n = 5. The control groups were retained under standard lighting. However, for the intervention groups, the lighting was manipulated as shown in Figure 1 between 8:00 am and 6:00 pm (Figure 1). All other conditions remained similar to the control groups’ circumstances. Daily interventions were uniform, and the modeling process continued for four weeks.

Throughout the modeling period, the body weights of the mice were measured and recorded weekly. After four weeks of intervention, the mice were humanely sacrificed at 8:00 a.m. using femoral artery bloodletting after anesthesia. Organs including the brain, liver, and kidneys were isolated. The organ weights were measured, after which the organ index was calculated (organ weight/body weight × 1%). The brain tissue was quickly frozen using liquid nitrogen and then stored at −80 °C for later transcriptome analysis. Blood samples were centrifuged at 3000 rpm for 12 min, with the resulting serum separated and conserved at −20 °C for subsequent examination of biochemical markers.

### 2.2. Analysis of Cerebral Index and Weight Gain Trends

By analyzing data from all four groups throughout the modeling stage, the average weight along with the standard deviation (AV ± SD, g) was calculated weekly for each group. The average cerebral index and the amount of weight gain for each group were computed, as well [22].

### 2.3. Testing and Analysis of Blood Biochemical Markers

The instrument used was a fully automatic biochemical analyzer (Mindray BS-800, Shenzhen, China). Biochemical indicators of interest included alanine aminotransferase (ALT), aspartate aminotransferase (AST), creatinine (Cr), serum urea nitrogen (BUN), reduced glutathione peptide (GSH), snd malondialdehyde (MDA), with corresponding reagents purchased from Beijing Solarbio Science & Technology Co., Ltd. (Beijing, China).

A 0.6 mL sample was taken for each of the gathered serum samples and diluted four-fold. Sample processing followed instructional guidelines for ALT, AST, Cr, BUN, GSH, and MDA testing. Subsequently, analysis was conducted with an automatic biochemical analyzer. The recorded results were then inputted into a calculation to obtain the group average (final result × 4).

### 2.4. Pathological Staining of Tissues

Samples with approximate dimensions of 5 mm × 5 mm × 2 mm were collected from the previously isolated liver, kidney, and brain tissues. After rinsing with physiological saline, the tissues were fixed in a 10% formalin solution and embedded in paraffin. Afterward, the liver, kidney, and brain tissues were refrigerated at 4 °C for 12 h. Embedded liver and kidney tissues were sectioned and stained using the hematoxylin–eosin (HE) method. Tissues were sliced to 4 μm thickness, processed, stained, sealed by neutral resin, and viewed under a microscope (200–400 times).

For neuronal analysis, Nissl staining and Aβ protein (1–40) staining were employed on brain tissue. The brain tissue was sectioned (6–8 μm), dewaxed, and hydrated. For Nissl staining, after rinsing with distilled water, sections were stained with a 12.5% toluidine blue solution. For staining of Aβ protein complexes, an Amyloid β 1–40 (Aβ1-40) staining kit (Shanghai FanTai Biotechnology Co., Ltd., Shanghai, China) was utilized. The sections were then mounted with neutral resin and observed microscopically (100–200 times).

### 2.5. Brain Tissue Transcriptome Testing and Analysis

The transcriptomic sequencing was outsourced to Wuhan MetWare Biotechnology Inc. (Wuhan, Hubei Province, China) From each of the following groups—normal–control, normal–circadian disruption, AD–control, and AD–circadian disruption—five brain tissue samples were randomly selected. After unifying the quantity, the total RNA was extracted, and mRNA strands with polyA tails were selectively isolated from the total RNA using Oligo(dT) magnetic beads according to the kit protocols (Solarbio). The mRNA strands were fragmented to serve as templates for synthesizing first-strand cDNA, to which buffer, dNTPs, and DNA Polymerase I were added for second-strand cDNA synthesis. This was followed by cDNA purification, end repair, adding A-tails, and ligation of sequencing adapters. PCR amplification was performed on fragments of appropriate sizes, and a cDNA library was established. After checking the library’s insert size and effective concentration in each case, libraries were combined based on the targets’ sequencing data volume, and Illumina platform sequencing was performed. The raw data were then filtered using fastp software (v0.23.4) to omit low-quality (Q ≤ 20) reads, reads containing sequencing adapters, and paired reads with over 10% unidentified bases (N). After assessing sequencing error rates and the GC content distribution, clean data were finally obtained. During the analysis, DESeq was used to identify differentially expressed genes between the groups. Reads on genes were computed and cumulated using featureCounts. Based on DESeq, the total number of differential genes and the count of upregulated and downregulated genes for each group were computed. Differential gene statistics graphs, volcano plots, heatmaps of differential expression clustering, Venn diagrams for differential genes, and scatter diagrams for multi-combination Gene Ontology (GO) enrichment of differential genes were generated from the obtained data.

### 2.6. Evaluation of mRNA m^6^A Regulation Level in Mice Brain Tissue

The method here was adapted from [23]. We obtained the total mRNA from mouse brain tissues, hydrolyzed it into nucleosides, and used LC-MS/MS to evaluate the m^6^A/A ratio, which indicates the level of m^6^A modification of the genes. In total, 0.1 g of brain tissue was taken from each group. After mixing with 1 mL of Trizol reagent (Invitrogen, Carlsbad, CA, USA), the total RNA was extracted and quantified using a NanoDrop One UV-Vis Spectrophotometer (Thermo Scientific, Waltham, MA, USA). Subsequent DNase I treatment and mRNA isolation were performed using DNase I, RNase free (Thermo Scientific) and a Dynabeads™ mRNA Purification Kit (Invitrogen), respectively, after which the mRNA concentration was measured using the spectrophotometer.

We fragmented 200 ng of mRNA from each group into mononucleosides using the method of Liu et al. [23]. After fragmentation, 4 μL of each solution was subjected to UPLC-MS/MS in the multiple reaction monitoring (MRM) mode. The m^6^A regulation level for each group was finally computed by dividing the area under the m^6^A peak by that under the adenine (A) peak (m^6^A/A).

### 2.7. Investigation of mRNA m6A Sites in the Gene Hif3α

*Hif3α* nucleotide sequence data were obtained from the website https://www.ncbi.nlm.nih.gov/nuccore/NM_ (accessed on 2 May 2024), and we screened for three probable sequences for m^6^A methylation—GGACU(T)/GAACU(T)/GAACA. Through an online database search on http://m6avar.renlab.org/, we gathered the details of all the *Hif3α* methylation sites, ranking them based on m^6^A source information. In the top three positions were chr19:46303617(+), chr19:46303617(+), and chr19:46303672(+). Upon integrating data on m^6^A-related variations and taking into account the human source requirement, chr19:46303617(+) (i.e., m^6^A site 3632) was chosen as the methylation site for further exploration. Building on base complementarity and reverse complementarity principles, methylation-specific primers were designed. Starting from the A base, both the sixth A base and the first preceding it (referred to as the N site) were selected for the design. Should methylation arise at the X site, its Ct value would exceed the N site’s Ct value, serving as a benchmark to predict the methylation status at the anticipated site 3632 of the *Hif3α* gene.

The mRNA extraction and subsequent reverse transcription were performed using a PrimeScript RT Reagent Kit (Perfect Real Time) (TaKaRa). We used 0.5 μg of total mouse RNA for each group. According to the kit’s instructions, a 10 μL reverse transcription system per group was established and incubated at 37 °C for 15 min, then terminated at 85 °C for 5 s and preserved at 4 °C. The SELECT qPCR process was as follows: each group contributed 2 μL of cDNA solution, to which we added 10 μL of 2× TB Green Fast qPCR mix (TaKaRa), 0.8 μL of X- and N-site primers (10 μM), 0.4 μL of 50× ROX Reference Dye II (TaKaRa), and sterile distilled water to bring the total volume of the SELECT qPCR system to 20 μL. The mix was briefly centrifuged and distributed in a 96-well plate, with three replications for each gene fragment detection in every group. Amplification was conducted on a 7500 FAST machine using a program of 30 s at 95 °C, followed by 40 cycles alternating 3 s at 95 °C with 12 s at 60 °C.

### 2.8. Western Blot Evaluation of HIF3A’s Roles in the Circadian Disruption Model

A volume calculated to contain 50 mg protein from brain tissue or cells was loaded with a 4:1 volume of 5× protein loading buffer (Solarbio), denatured at 100 °C for 15 min, incubated on ice for 3 min, centrifuged at 4 °C and 13,000 rpm for 15 min, and refrigerated at −20 °C. For SDS-PAGE, 1× Tris-glycine electrophoresis buffer was used to fill the electrophoresis unit (Beijing Liuyi Biotechnology Co., Ltd., Beijing, China), and samples and pre-stained rainbow protein marker (Solarbio) were loaded onto the gel plates and electrophoresed initially at 100 V for 20 min, then at 130 V for about 40 min until the protein marker was completely separated.

To quantify the protein expression levels of HIF3A-associated proteins KDM3A and TGF-β1, with respect to the control protein GAPDH within brain tissue, WB was performed. Millipore PVDF membranes were prepared through a brief, one-minute immersion in methanol. The membranes, sponges, and filter paper were submerged in a 1× transfer buffer kept at 4 °C. The assembly within the transfer apparatus was organized from the negative to the positive pole as follows: sponge, filter paper, gel, membrane, filter paper, and sponge. The membranes then underwent a 70 V transfer for two hours. After the transfer, the membranes were rinsed with water and incubated for an hour in 10% skimmed milk on a shaking platform. They were then washed using 1× TBST with 10 min of low-speed shaking. The membranes were further incubated with a cocktail of rabbit anti-HIF-3α, KDM3A, and TGF-β1 antibodies (Santa Cruz, CA, USA) in combination with 1× TBST (Solarbio), mixed in a 1:1000 ratio, for two hours at 25 °C on a shaking platform. The membranes were washed five times using TBST, for 5 min each time, and next they were incubated with a 1:3000 mixture of goat anti-rabbit IgG-HRP and 1× TBST for an hour. After another five rounds of TBST washing, the membrane was washed five times with distilled water. The ECL developer (Solarbio) for the membrane was prepared in darkness and added to the membrane. Chemiluminescence signals were captured with a designated imaging system, and the relative amounts of HIF3A, KDM3A, and TGF-β1 proteins in each group of mouse brain tissues were quantified concerning GAPDH expression.

### 2.9. Protein Detection of the Hif3α/KDM3A/TGF-β1 Axis in PC12 Cells and N2a Cells

Pheochromocytoma cells (PC12) and neuro-2a cells (N2a) were purchased from the American Type Culture Collection (ATCC, Manassas, VA, USA), and culturing medium, serum, and antibiotics were purchased from Solarbio. PC12 cells were cultured with DMEM containing 10% fetal bovine serum, 5% horse serum, and 1% penicillin/streptomycin in 6-well plates, which were cultivated at 37 ℃ in a 5% CO_2_ incubator. N2a cells were cultured with MEM containing 10% fetal bovine serum and 1% penicillin/streptomycin in 6-well plates, which were cultivated at 37 ℃ in a special incubator (5% CO_2_, 74% N_2_, and 21% O_2_). When the cell intensity reached about 80%, Aβ1-42 solution (final concentration was 20 μM) was added to the wells, and the in vitro AD model construction was completed after 48 h. After the detection of Aβ1-42 proteins in the two types of cells, we confirmed that the models were successfully constructed. Then, we utilized the Hif3α SiRNA fragment to treat the cells for 24 h, and the expression levels of Hif3α/KDM3A/TGF-β1 proteins were measured by wb, to investigate the effect of Hif3α on the expression of KDM3A and TGF-β1 in the present model.

### 2.10. Statistical Analysis

All data, presented as the average ± standard deviation (AV ± SD), were analyzed using SPSS 19.0 and GraphPad Prism 7.0 (GraphPad Software Inc., San Diego, CA, USA). The control and intervention group data were analyzed by *t*-tests, with the significance threshold set at *p* < 0.05.

## 3. Results

### 3.1. Disruption of Circadian Rhythms Influences Weight Gain and Cerebral Index in Normal and AD Mice

**Figure 2:**
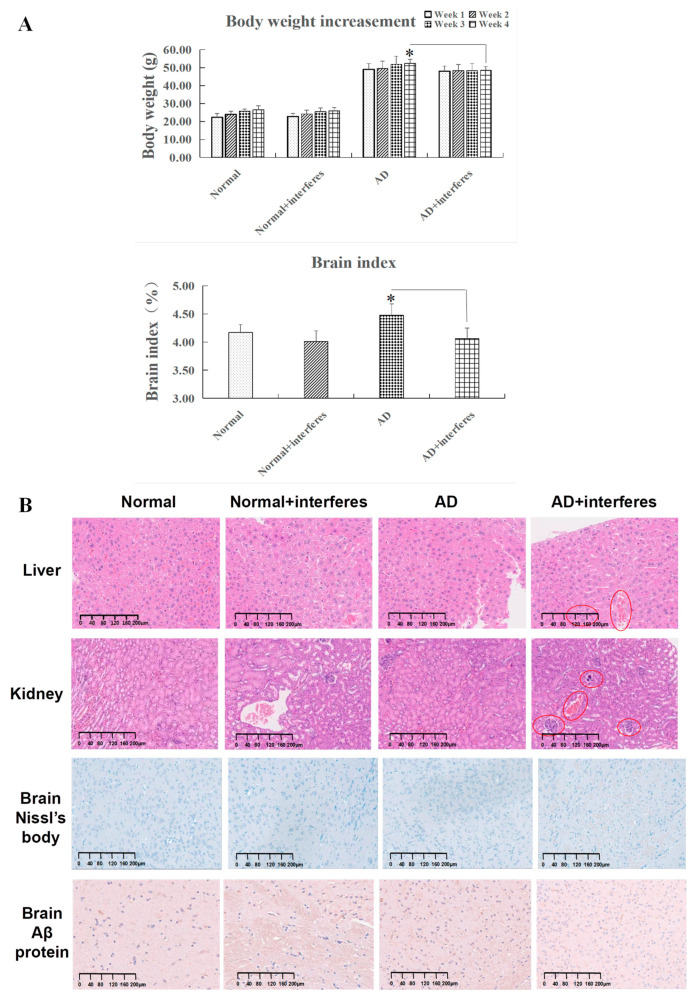
Mice body weight gain, cerebral index, and special staining. (**A**) Variations in body weight and cerebral indexes across four mouse cohorts. (**B**) Results of histopathological staining of liver and kidney sections after hematoxylin and eosin (HE) staining and brain sections after Nissl body and Aβ protein complex staining in cerebral cortex. All images are displayed at a magnification of 200×. n = 5. The asterisk (*) indicates significant statistical differences when comparing two groups, with a *p*-value of less than 0.05.

Considering the link between circadian rhythm disruption and metabolic imbalances and neurodegenerative diseases, this study examined trends in the mice’s body weight gain and cerebral indexes (Figure 2A). While all mice experienced weight gain, the rate of change in weight steadily decreased over time in the normal and AD mice under an intervention in comparison to the respective control groups. The AD–intervention group displayed the least weight gain. By week four, compared to the AD–control group, a declining weight gain trend was observed in the AD–intervention group (*p* < 0.05), reinforcing the conclusion that circadian rhythm disruption impaired AD mice’s physical function and homeostatic balance. In addition, the cerebral indexes in both the normal and AD intervention groups were significantly lower than in their non-intervention counterparts. This decrease was especially significant in the AD intervention group compared to the control AD group (*p* < 0.05), indicating that circadian rhythm disruption harmed the brain’s capabilities of information processing and behavioral control (Figure 2A).

### 3.2. HE Staining and Special Marker Staining Reveal the Damage to Mice’s Liver, Kidney, and Brain Tissues from Circadian Disruption

HE staining clearly revealed histopathological contrasts between the control and intervention groups in mouse liver and kidney tissues. As in Figure 2B, compared with the other groups, liver tissues in the AD intervention groups (in red circles) experienced more bleeding instances, occasional edema, and obvious cytomorphosis. Kidney tissues of the normal– and AD–intervention groups displayed slight hemostasis, while the AD–intervention group had aggregations of inflammatory cell nuclei (shown in red circles), signifying potential inflammation. These findings suggested that circadian rhythm disruption provoked liver and kidney damage through bleeding, edema, and inflammation (Figure 2B).

For brain tissues, toluidine blue staining highlighted changed in Nissl bodies in the cerebral cortex within the four mice cohorts (Figure 2B). After circadian rhythm disruption, both normal and AD mice demonstrated significantly fewer Nissl bodies than the control groups. In the intervention groups, Nissl bodies appeared fragmented, implying possible proliferation due to stress or structural fragmentation. This observation was most pronounced in AD mice post-intervention.

The staining of brain Aβ protein complexes in the cerebral cortex revealed their deposition in the brain tissue of the four cohorts (Figure 2B). Both normal and AD mice under circadian rhythm disruption exhibited significantly more Aβ protein deposition in brain tissue than that in corresponding control groups, with the AD–intervention group showing the largest amount of deposition. Notably, some Aβ protein depositions in the normal–intervention group appeared relatively concentrated. These phenomena indicated that circadian rhythm disruption could impair brain functioning in both normal and AD communities.

### 3.3. Biochemical Marker Analysis Reveals Damage to Mice’s Liver, Kidney, and Antioxidative Capacities from Circadian Disruption

**Figure 3:**
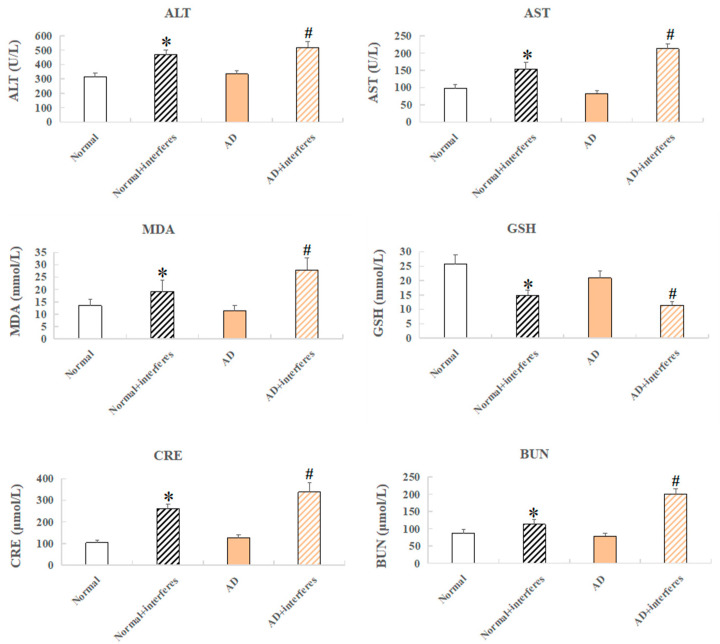
Mice serum biochemical markers. Biochemical indicators include ALT, AST, CRE, BUN, GSH, and MDA. n = 5. An asterisk (*) indicates a significant statistical difference from the normal groups (*p* < 0.05), while an octothorpe (#) identifies a significant difference compared to the AD–control group (*p* < 0.05).

Mouse serum samples were analyzed for biomarkers including alanine aminotransferase (ALT), aspartate aminotransferase (AST), creatinine (Cr), serum urea nitrogen (BUN), reduced glutathione peptide (GSH), and malondialdehyde (MDA) (Figure 3). ALT and AST, liver function markers, were significantly elevated in both the normal– and AD–intervention groups (*p* < 0.05), confirming the finding of liver damage from a disrupted circadian rhythm based on previous HE staining (Figure 3). Increased MDA, an indication of oxidative stress from cell membrane lipid peroxidation, and lowered GSH, an antioxidant with integrated detoxification effects, were detected in the intervention groups, which revealed increased oxidative stress and lowered antioxidant capabilities, respectively, due to circadian disruption (in comparison with the control groups, *p* < 0.05). The intervention groups also exhibited higher levels of creatinine—usually filtered out by the kidneys, which indicated kidney damage—and raised urea nitrogen concentrations, reflecting impaired kidney functioning compared to the control groups (Figure 3).

### 3.4. Investigation and Analysis of Transcription Differentials in Brain Tissue

**Figure 4:**
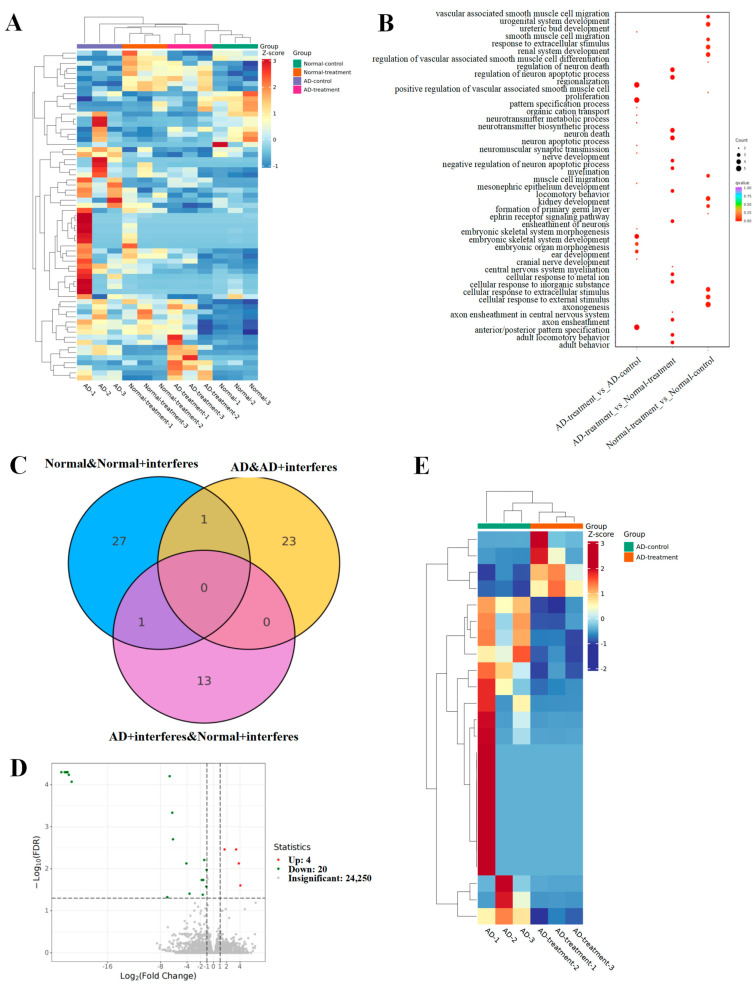
Analysis of mouse brain tissue transcriptomic patterns. (**A**) Heatmap for the expression of differential genes. (**B**) GO enrichment analysis of brain-function-related differential genes. (**C**) Venn diagram of differential genes. (**D**) Volcano plot of differential gene expression. (**E**) Clustering heatmap of key candidate genes.

To delve into how circadian disruptions impacted normal and AD mice on the gene expression level, as well as to select sensitive key genes for further investigation, we tested and analyzed mice brain tissue transcriptomes from each group. The outcomes are depicted in Figure 4.

The heatmap (Figure 4A) represents the total differential genes. Cooler hues signify a reduction in the expression levels of specific genes in the intervention group versus the control group, while warmer colors indicate an upsurge in expression. The greater the contrast between two colors, the greater the difference in gene expression level. The gene ontology (GO, Figure 4B) enrichment examination of differential genes provided insights into the biological processes that these genes impact. The dots’ magnitudes indicated their relevance to these processes, with larger dots signaling a stronger relevance. Genes associated with neuronal apoptosis regulation or myelin formation had greater prevalences in both AD– and normal–intervention mice. The Venn diagram (Figure 4C) illuminated the unique and shared differential genes among various group comparisons. Specifically, we observed 29 unique differential genes in normal–control mice and normal–intervention mice and 24 in the AD–control mice in comparison with the AD–intervention mice (Figure 4D). Fourteen unique genes emerged when comparing the AD–intervention mice to the normal–intervention mice, and an overlapping analysis of both the normal/normal–intervention group and the AD/AD–intervention group unveiled one shared differential gene (Figure 4E).

### 3.5. Identification of m^6^A Methylation Sites within the Hif3α Gene and Associated Signaling Pathways of the HIF3A Protein in Brain Tissue

**Figure 5:**
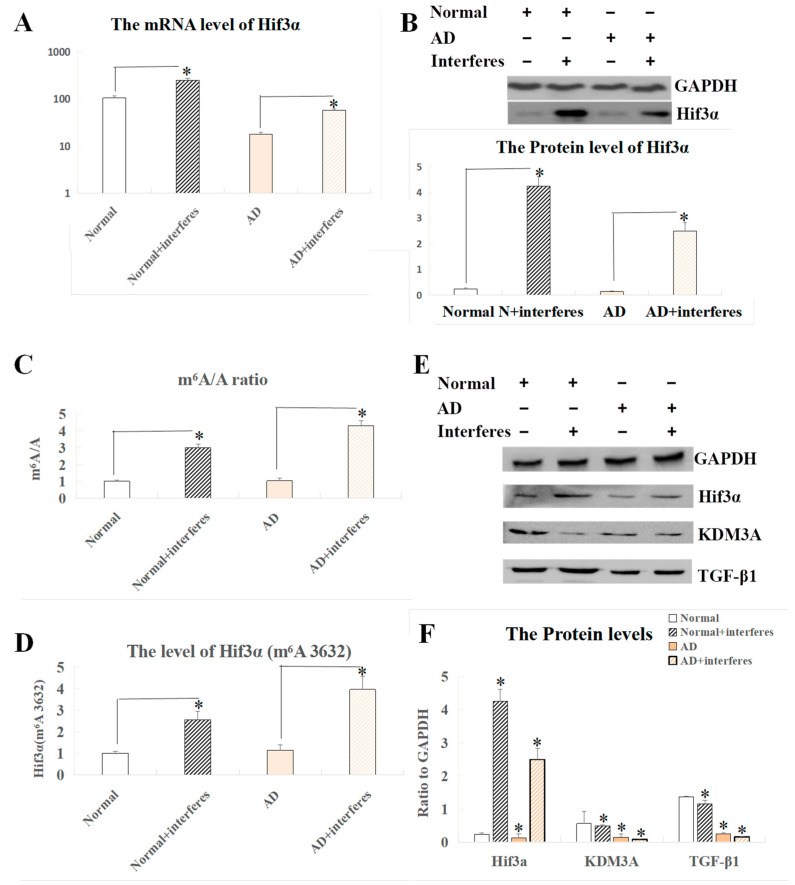
Analysis of *Hif3α* mRNA m^6^A and Western blot results of related proteins in brain tissue. (**A**) Expression level of the *Hif3α* gene at the mRNA level. (**B**) Protein-level expression of the *Hif3α* gene. (**C**) m^6^A/A ratio of *Hif3α* mRNA. (**D**) m^6^A level at *Hif3α* site 3632. (**E**) Western blot results of HIF3A and functionally related proteins. (**F**) Quantitative representation of HIF3A and functionally related proteins with respect to GAPDH. The asterisk (*) indicates significant statistical differences compared to normal mice, *p* < 0.05. n = 5.

In screening and co-analysis of the sensitive genes in brain tissue, a markedly increased expression of the *Hif3α* gene was observed in the normal– and AD–intervention groups compared to the respective control groups (*p* < 0.05). Past studies on the role of this gene and related clusters (*HIF1* and *HIF2*) in stroke instances arising from brain ischemia suggested that the HIF3A protein could have similar functions to Hif1 and Hif2 proteins, thus possibly becoming crucial in brain damage experimental models. Our measurements of HIF3A expression at both the mRNA and protein levels in brain tissue indicated a largely consistent expression pattern (Figure 5A,B), showing that there was a significant surge in the expression of this factor within the interventions relative to the control group (*p* < 0.05). Figure 5C–F exhibit our analysis of the *Hif3α* m^6^A methylation site (3632) and expression levels of HIF3A-related proteins. Both the normal– and AD–intervention groups demonstrated a significant rise in the m^6^A/A quotient of *Hif3α* mRNA as compared to the respective non-intervention groups (*p* < 0.05, Figure 5C). This result confirmed a critical epigenetic modification at the m^6^A level in mice with disrupted circadian rhythms. The definitive m^6^A location within the gene’s sequence was pinpointed at site 3632 (Figure 5D), which was ascertained to be a key player in the post-intervention elevation of HIF3A protein expression seen in the current experimental model, indicating its correlation with AD progression. Complementing the previously mentioned substantial trend of increased expression of HIF3A protein in the intervention groups vs. the control groups, additional probing involved comparing the expression levels of the functionally related KDM3A and TGF-β1 proteins across the brain tissues of the four mice cohorts. Our findings highlighted the lowered presence of KDM3A and TGF-β1 within the intervention groups when contrasted with the respective controls, with this phenomenon more significant in AD mice as compared to normal mice (*p* < 0.05, Figure 5E,F).

### 3.6. Identification of the Hif3α/KDM3A/TGF-β1 Axis in PC12 Cells and N2a Cells

**Figure 6:**
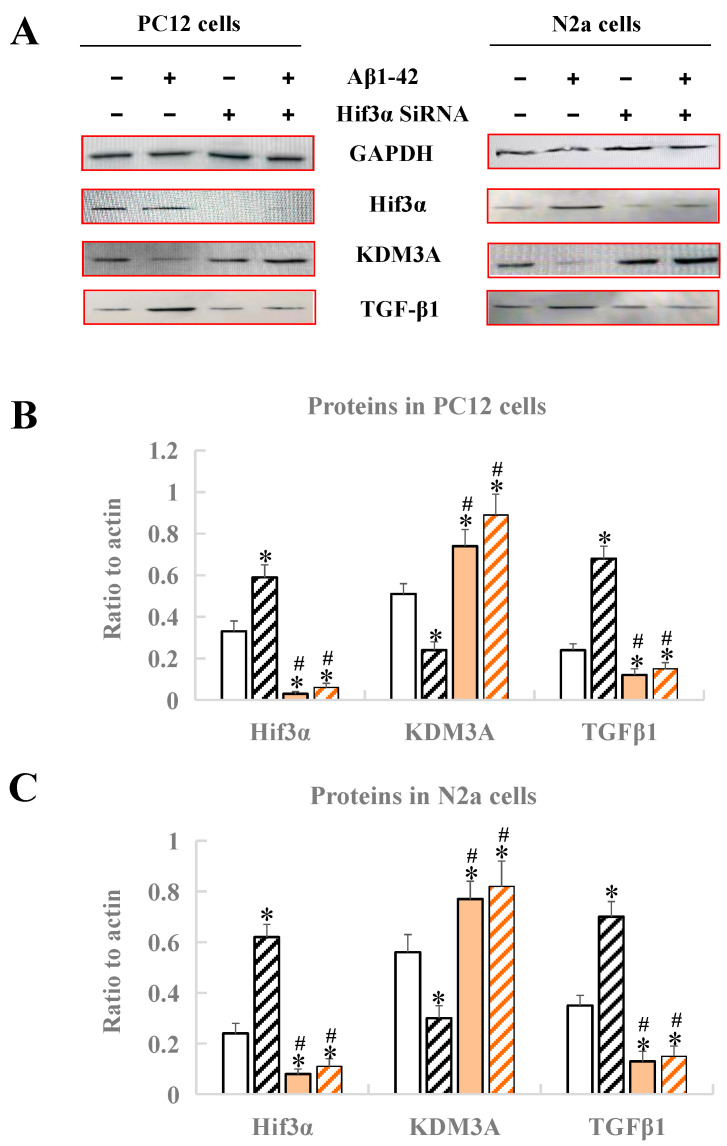
Analysis of protein expression by Western blotting in PC12 cells and N2a cells. (**A**) Western blot bands of four proteins. (**B**) Protein expression of Hif3α/KDM3A/TGF-β1 in PC12 cells. (**C**) Protein expression of Hif3α/KDM3A/TGF-β1 in N2a cells. * Significant statistical differences compared to the control group without any treatment, *p* < 0.05. ^#^ Significant statistical differences compared to the *Hif3α* SiRNA group, *p* < 0.05. n = 3.

After treatment with Aβ1-42 and *Hif3α* SiRNA, the expression levels of *Hif3α*/KDM3A/TGF-β1 in PC12 cells and N2a cells were measured by Western blotting. As Figure 6 demonstrates, no matter whether in PC12 cells or in N2a cells, the protein levels of Hif3α and TGFβ1 obviously increased, and the protein level of KDM3A decreased, when compared with the control (*p* < 0.05), indicating that the in vitro AD model was successfully constructed (Figure 6). After the treatment of the *Hif3α* SiRNA fragment, the protein levels of Hif3α and TGFβ1 were significantly upregulated, and that of KDM3A was downregulated when compared with the control (*p* < 0.05), further proving that KDM3A and TGFβ1 were the downstream factors of Hif3α, and that the *Hif3α*/KDM3A/TGF-β1 axis might play key roles in the progression of AD caused by circadian rhythm disruption (Figure 6). 

## 4. Discussion

The current literature on the effects of circadian rhythms on the progression of AD mainly focuses on chronic metabolic diseases, inflammatory responses, cellular oxidative stress, and aging caused by abnormal expression of circadian rhythm genes. Through constructing a model of circadian rhythm disruption in animals, the present study analyzed and compared weight changes, cerebral indexes, biochemical markers, and gene expression differences in AD model mice (APP/PS1) and normal mice (C57BL/6N), preliminarily screened out candidate key genes, and validated the important role of the key gene *Hif3α* at the molecular level of epigenetics in this model.

As the results demonstrated, this study preliminarily found that circadian rhythm disruption could accelerate disease progression in the AD model, as illustrated in the reduced weight gain, liver and kidney impairment, heightened oxidative stress, reduced antioxidant capacity, and neural damage in the mice models. Contrary to prior reports suggesting that circadian disruption leads to weight gain [24], the mice under the circadian disruption intervention in our study showed a decreased weight gain. This may have been due to the consistent, not intermittent, circadian rhythm disruption, with the consistent rhythm disruption intervention potentially leading to increased anxiety and irritation levels in mice. Nevertheless, our model was proven effective as compared with the control groups. Mice in the intervention groups had signs of liver and kidney damage, including occasional tissue bleeding, swelling, and inflammation (Figure 2B), and abnormal levels of biochemical markers (Figure 3). Furthermore, brain function impairment in the intervention groups was marked by a decreased cerebral index (Figure 2A), possible Nissl body proliferation or fragmentation (Figure 2B), and increased expression levels of genes with functions tied to neuronal death, apoptosis, and myelin formation (Figure 4B). Synthesizing these results, we suggested that circadian disruption impaired the metabolic and nervous systems in mice, fostered malignant AD progression, and increased the dementia risk in normal mice. The findings underscored the need to unravel the molecular mechanisms relating circadian disruption to AD’s accelerated progression.

Besides the above phenotypic experimental results, we also for the first time obtained the following findings of how circadian rhythm disruption accelerates AD progression. Both C57BL/6N mice and APP/PS1 double-transgenic mice with disrupted circadian rhythms exhibited significant elevation in HIF3A expression in brain tissue compared to the control groups; moreover, m^6^A regulation of the *Hif3α* gene was found to be influenced by circadian rhythm disturbances, specifically situated at *Hif3α* site 3632.

HIF3A, or hypoxia-inducible factor 3, alpha subunit, is a member of the hypoxia-inducible factor (HIF) family, which guides hypoxia-response gene expression in various tissues. Hypoxia-inducible factors are heterodimers mediating tissue hypoxia responses, composed of an oxygen-sensitive α subunit and a stably expressed β subunit [25]. HIF1A, HIF2A, and HIF3A are the α subunits in mammals, and they all can dimerize with HIF1B (Aryl Hydrocarbon Receptor Nuclear Translocator, ARNT) via their HLH and PAS domains. These dimers bind to genome hypoxia response elements (HREs) to promote gene regulation. Few studies have focused on HIF3A due to its unique structure and numerous variants compared to HIF1A and HIF2A [26]. However, in one study, its associated biological processes were discovered to include angiogenesis, cell apoptosis, RNA polymerase II transcription regulation, and hypoxia responses. Furthermore, anomalies in HIF3A regulation were linked to maladies including inflammatory responses [27], adipose tissue dysfunction [28], stroke [29], and cancer [30]. Importantly, our study complements earlier limited literature [30,31], further confirming the potential of upregulated HIF3A expression for driving the AD risk and progression. Moreover, considering that the increased *Hif3α* gene expression following stroke was shown to contribute to the hypoxia response, inflammation, and angiogenesis [32], stroke patients and their families might have a heightened AD risk, providing a reference for AD screening and prevention. Through analysis of transcription differentials, this study found that the *Hif3α* gene might be key in the progression of AD in a circadian rhythm disruption model.

Considering the relationship between m^6^A regulation and Alzheimer’s disease, previous studies have found a markedly low expression of the m^6^A writer METTL3 in the hippocampus in AD patients’ brain RNA libraries [13]. They confirmed this on the protein level in post-mortem human brain tissue samples, and observed METTL3 protein accumulation in insoluble components, which correlated with insoluble tau protein levels in the samples [13]. Similarly, Zhao et al. identified decreased METTL3 expression and significantly diminished m^6^A levels in the hippocampi of AD patients’ brains [33]. They linked m^6^A modification to memory deficits, synapse loss, neuron death, oxidative stress, and cell cycle irregularities in brain cells; in addition, they found that increasing the concentrations of Aβ oligomers decreased METTL3 expression, but METTL3 overexpression could ameliorate Aβ-induced synaptic damage and cognitive disorders [33]. Li et al. examined pro-inflammatory M1-L, anti-inflammatory M2-L, and resting M0-L phenotypes in primary cultured rat microglial cells [34]. They found an increased expression of some m^6^A-modified genes during the transition from an M0-L to an M1-L phenotype, indicating their potential involvement in inflammatory responses in neurodegenerative conditions [34]. This study examined the m^6^A levels in the total brain tissue of C57BL/6N mice and APP/PS1 mice post circadian disruptions. Both AD mice and normal mice in the intervention groups had a significant increase in m^6^A/A levels compared to their control groups. Coupled with transcriptome sequencing analysis, we proved that the *Hif3α* gene exhibited heightened expression levels. By evaluating m^6^A methylation sites of *Hif3α* via online databases, and using SELECT-qPCR to confirm site 3632 as the specific regulation site in our circadian disruption mouse model, we advanced the understanding initiated by prior findings relating increased HIF3A expression to AD [31,32]. We highlighted a mechanism where circadian disruptions impacted m^6^A methylation at *Hif3α* site 3632, promoted HIF3A expression, and potentially sped up AD progression or increased the AD risk. When considered alongside observations of circadian rhythm disruptions’ impact on mice’s bodily and neural functions, this mechanism steered critical contributions to AD progression.

Furthermore, to validate the proposed mechanism above, we selected protein factors with functions associated with HIF3A and conducted Western blot experiments in mice brain tissues and two types of cells (PC12 and N2a), further validating that under circadian rhythm disruption, the malignant progression of AD correlated with *Hif3α*’s RNA m^6^A methylation at position 3632. Moreover, we found an increase in HIF3A expression accompanied by a decrease in KDM3A protein and an increase in TGF-β1 protein. If we treated the cells with *Hif3α*’s siRNA, we found that the KDM3A protein was expressed more and the TGF-β1 protein was expressed less immediately (*p* < 0.05), while the HIF3A protein directly regulated the expression levels of KDM3A protein (opposite direction) and TGF-β1 protein (same direction), proving that the three proteins have a shared pathway under circadian rhythm disruption in AD, i.e., an Hif3α/KDM3A/TGF-β1 axis. Since both KDM3A and TGF-β1 had protective roles under hypoxic organ conditions and contributed to responses to neuroinflammation, our research proved that circadian rhythm disruption accelerates the progression of AD via *Hif3α* m^6^A methylation at position 3632, which modulates downstream KDM3A and TGF-β1. 

## 5. Conclusions

To conclude, we have verified that long-term circadian rhythm disruption will accelerate the progression of AD, mainly by regulating *Hif3α* m^6^A methylation at site 3632 and affecting the expression of the Hif3α/KDM3A/TGF-β1 axis. Furthermore, as HIF3A, KDM3A, and TGF-β1 seem linked to hypoxic conditions and AD progression, further studies are required to elucidate the links between circadian rhythm disruptions of different types, neuroinflammation-related diseases (including AD and brain hypoxia), and special gene m^6^A methylation modifications.

## Data Availability

Data generated for this study are included in the manuscript.

## References

[1] Patke A., Young M.W., Axelrod S. (2020). Molecular mechanisms and physiological importance of circadian rhythms. Nat. Rev. Mol. Cell Biol..

[2] Qian J., Block G.D., Colwell C.S., Matveyenko A.V. (2013). Consequences of exposure to light at night on the pancreatic islet circadian clock and function in rats. Diabetes.

[3] Turek F.W., Joshu C., Kohsaka A., Lin E., Ivanova G., Mcdearmon E., Laposky A., Losee-Olson S., Easton A., Jensen D.R. (2005). Obesity and metabolic syndrome in circadian *clock* mutant mice. Science.

[4] Chellappa S.L., Vujovic N., Williams J.S., Scheer F.A.J.L. (2019). Impact of circadian disruption on cardiovascular function and disease. Trends Endocrinol. Metab..

[5] Zhou L., Zhang Z., Nice E., Huang C., Zhang W., Tang Y. (2022). Circadian rhythms and cancers: The intrinsic links and therapeutic potentials. J. Hematol. Oncol..

[6] De-Paula V.J., Radanovic M., Diniz B.S., Forlenza O.V. (2012). Alzheimer’s disease. Sub-Cell. Biochem..

[7] Breijyeh Z., Karaman R. (2020). Comprehensive review on alzheimer’s disease: Causes and treatment. Molecules.

[8] Meyer K.D., Jaffrey S.R. (2017). Rethinking m^6^a readers, writers, and erasers. Annu. Rev. Cell Dev. Biol..

[9] Fustin J., Doi M., Yamaguchi Y., Hida H., Nishimura S., Yoshida M., Isagawa T., Morioka M.S., Kakeya H., Manabe I. (2013). Rna-methylation-dependent rna processing controls the speed of the circadian clock. Cell.

[10] Wang C., Yeh J., Shie S., Hsieh I., Wen M. (2015). Circadian rhythm of rna n6-methyladenosine and the role of cryptochrome. Biochem. Biophys. Res. Commun..

[11] Meyer K.D., Saletore Y., Zumbo P., Elemento O., Mason C.E., Jaffrey S.R. (2012). Comprehensive analysis of mrna methylation reveals enrichment in 3′ utrs and near stop codons. Cell.

[12] Leonetti A.M., Chu M.Y., Ramnaraign F.O., Holm S., Walters B.J. (2020). An emerging role of m6a in memory: A case for translational priming. Int. J. Mol. Sci..

[13] Huang H., Camats-Perna J., Medeiros R., Anggono V., Widagdo J. (2020). Altered expression of the m6a methyltransferase mettl3 in alzheimer’s disease. eNeuro.

[14] Cermakian N., Lamont E.W., Boudreau P., Boivin D.B. (2011). Circadian clock gene expression in brain regions of alzheimer’s disease patients and control subjects. J. Biol. Rhythm..

[15] Musiek E.S., Bhimasani M., Zangrilli M.A., Morris J.C., Holtzman D.M., Ju Y.S. (2018). Circadian rest-activity pattern changes in aging and preclinical alzheimer disease. JAMA Neurol..

[16] Qin L., Xu Y., Yu X., Toneff M.J., Li D., Liao L., Martinez J.D., Li Y., Xu J. (2017). The histone demethylase kdm3a is required for normal epithelial proliferation, ductal elongation and tumor growth in the mouse mammary gland. Oncotarget.

[17] Lodyga M., Hinz B. (2020). Tgf-β1-a truly transforming growth factor in fibrosis and immunity. Semin. Cell Dev. Biol..

[18] Liu K., Zhu R., Jiang H., Li B., Geng Q., Li Y., Qi J. (2022). Taurine inhibits kdm3a production and microglia activation in lipopolysaccharide-treated mice and bv-2 cells. Mol. Cell. Neurosci..

[19] Doyle K.P., Cekanaviciute E., Mamer L.E., Buckwalter M.S. (2010). Tgfβ signaling in the brain increases with aging and signals to astrocytes and innate immune cells in the weeks after stroke. J. Neuroinflamm..

[20] Guo X., Zhang B., Zhang J., Liu G., Hu Q., Chen J. (2022). The histone demthylase kdm3a protects the myocardium from ischemia/reperfusion injury via promotion of ets1 expression. Commun. Biol..

[21] Yu Y., Li J., Zhou H., Xiong Y., Wen Y., Li H. (2018). Functional importance of the tgf-β1/smad3 signaling pathway in oxygen-glucose-deprived (ogd) microglia and rats with cerebral ischemia. Int. J. Biol. Macromol..

[22] Kumar V., Yadav E. (2021). Assessment of body weight, brain weight and neuro-somatic index in albino rat under toxic stress of lambda cyhalothrin and its modulation by green tea. J. Exp. Zool. India.

[23] Liu R., Zhao F., Wei J., Yu P., Zhang J., Wang Y., Li Z., Zhang J., Zhang X., Tian X. (2019). Determination of five nucleosides by lc-ms/ms and the application of the method to quantify *n*^6^-methyladenosine level in liver messenger ribonucleic acid of an acetaminophen-induced hepatotoxicity mouse model. J. Sep. Sci..

[24] Altaha B., Heddes M., Pilorz V., Niu Y., Gorbunova E., Gigl M., Kldigrewe K., Oster H., Haller D., Kiessling S. (2022). Genetic and environmental circadian disruption induce weight gain through changes in the gut microbiome. Mol. Metab..

[25] Majmundar A.J., Wong W.J., Simon M.C. (2010). Hypoxia-inducible factors and the response to hypoxic stress. Mol. Cell.

[26] Duan C. (2016). Hypoxia-inducible factor 3 biology: Complexities and emerging themes. Am. J. Physiol.-Cell Physiol..

[27] Cuomo F., Coppola A., Botti C., Maione C., Forte A., Scisciola L., Liguori G., Caiafa I., Ursini M.V., Galderisi U. (2018). Pro-inflammatory cytokines activate hypoxia-inducible factor 3α via epigenetic changes in mesenchymal stromal/stem cells. Sci. Rep..

[28] Pfeiffer S., Krueger J., Maierhofer A., Boettcher Y., Kloeting N., El Hajj N., Schleinitz D., Schoen M.R., Dietrich A., Fasshauer M. (2016). *hypoxia*-*inducible factor 3a* gene expression and methylation in adipose tissue is related to adipose tissue dysfunction. Sci. Rep..

[29] Luo J., Chen D., Mei Y., Li H., Qin B., Lin X., Chan T.F., Lai K.P., Kong D. (2023). Comparative transcriptome findings reveal the neuroinflammatory network and potential biomarkers to early detection of ischemic stroke. J. Biol. Eng..

[30] Wei L., Yuan N., Chen Y., Gong P. (2021). Aberrant expression of hif3a in plasma of patients with non-small cell lung cancer and its clinical significance. J. Clin. Lab. Anal..

[31] Kumar H., Lim J., Kim I., Choi D. (2015). Differential regulation of hif-3α in lps-induced bv-2 microglial cells: Comparison and characterization with hif-1α. Brain Res..

[32] Wang L., Cao J., Xu Q., Lu X., Yang X., Song Q., Chen S., Du K., Huang R., Zou C. (2021). 2-dodecyl-6-methoxycyclohexa-2,5-diene-1,4-dione ameliorates diabetic cognitive impairment through inhibiting hif3α and apoptosis. Front. Pharmacol..

[33] Zhao F., Xu Y., Gao S., Qin L., Austria Q., Siedlak S.L., Pajdzik K., Dai Q., He C., Wang W. (2021). Mettl3-dependent rna m6a dysregulation contributes to neurodegeneration in alzheimer’s disease through aberrant cell cycle events. Mol. Neurodegener..

[34] Li Q., Wen S., Ye W., Zhao S., Liu X. (2021). The potential roles of m6a modification in regulating the inflammatory response in microglia. J. Neuroinflamm..

